# Producing natural functional and low-carbon milk by regulating the diet of the cattle—The fatty acid associated rumen fermentation, biohydrogenation, and microorganism response

**DOI:** 10.3389/fnut.2022.955846

**Published:** 2022-10-19

**Authors:** Xiaoge Sun, Yue Wang, Xiaoyan Ma, Shengli Li, Wei Wang

**Affiliations:** ^1^State Key Laboratory of Animal Nutrition, Beijing Engineering Technology Research Center of Raw Milk Quality and Safety Control, College of Animal Science and Technology, China Agricultural University, Beijing, China; ^2^Animal Production Systems Group, Wageningen University & Research, Wageningen, Netherlands

**Keywords:** dietary fat, rumen fermentation, methane, biohydrogenation, microorganism

## Abstract

Conjugated linoleic acid (CLA) has drawn significant attention in the last two decades for its various potent beneficial effects on human health, such as anticarcinogenic and antidiabetic properties. CLA could be generally found in ruminant products, such as milk. The amount of CLA in ruminant products mainly depends on the diet of the animals. In general, the fat content in the ruminant diet is low, and dietary fat supplementation can be provided to improve rumen activity and the fatty acid (FA) profile of meat and milk. Especially, dietary 18-carbon polyunsaturated FA (C18 PUFA), the dominant fat source for ruminants, can modify the milk FA profile and other components by regulating the ruminal microbial ecosystem. In particular, it can improve the CLA in milk, intensify the competition for metabolic hydrogen for propionate producing pathways and decrease methane formation in the rumen. Therefore, lipid supplementation appears to be a promising strategy to naturally increase the additional nutritional value of milk and contribute to lower methane emissions. Meanwhile, it is equally important to reveal the effects of dietary fat supplementation on rumen fermentation, biohydrogenation (BH) process, feed digestion, and microorganisms. Moreover, several bacterial species and strains have been considered to be affected by C18 PUFA or being involved in the process of lipolysis, BH, CLA, or methane emissions. However, no review so far has thoroughly summarized the effects of C18 PUFA supplementation on milk CLA concentration and methane emission from dairy cows and meanwhile taken into consideration the processes such as the microorganisms, digestibility, rumen fermentation, and BH of dairy cattle. Therefore, this review aims to provide an overview of existing knowledge of how dietary fat affects rumen microbiota and several metabolic processes, such as fermentation and BH, and therefore contributes to functional and low-carbon milk production.

## Introduction

There is an increasing awareness of the relationship between health and diet among consumers, which leads to the nutritional quality of food becoming a hot topic. Functional foods, defined as foods or food components that have positive effects on human health beyond the basic nutritive value (1) became more and more popular in the market. Conjugated linoleic acid (CLA) is one of these functional food components, which was usually found in milk and ruminant meat. According to the study, about 70% of the CLA intake of American people is origin from dairy products (2). CLA has been shown to exert various potent beneficial effects on humans such as anticarcinogenic and antidiabetic properties (3, 4). Therefore, improving the biosynthesis of CLA in dairy cows is expected to have a contribution to the nutritive value of dairy products and subsequently human health.

Dietary fatty acid (FA) supplementation has become a widely accepted dietary strategy for improving the quality of animal products (5–7). There are two main categories of FAs: unsaturated fatty acids (UFAs) that consist of one or more double-carbon bonds and saturated fatty acids (SFAs) that lack double-carbon bonds. The UFA family contains monounsaturated fatty acids (MUFAs) having one double bond and polyunsaturated fatty acid (PUFA) with more than one double bond. Fat represents less than 5% of the total dry matter (DM) in most ruminant diets, with linoleic acid (LA; C18:2) dominating in forages and α-linolenic acid (ALA; C18:3) being more prevalent in concentrates (8).

Originally, fat supplementation is used to improve the energy values of diets to meet the energy requirements of dairy cows but it is also found to have additional functions to modify the FA profiles of meat (9) and milk (10) of the ruminants. Especially, C18 PUFA could increase the CLA in animal products. However, fat supplementation in ruminant diets, especially for C18 PUFAs, is limited due to its adverse effect on ruminal bacterial growth (11). In turn, ruminal microorganisms typically hydrogenate C18 PUFAs (12), which is considered as a detoxifying adaptation, and produce CLA (13, 14). This biohydrogenation (BH) process comprises several steps and pathways, depending on the PUFAs form, diet composition, and ruminal environment (15). Particularly, it can change the competition for metabolic hydrogen between propionate and methane production pathways (16–18). This suggests that fat supplementation can shift rumen function and reduce methane emissions (19). Methane plays a key role in anthropogenic climate change and its global warming potential is 298 times higher than carbon dioxide (20). It means that the addition of fat has a potential to contribute to milk CLA concentrate and greenhouse gas mitigation simultaneously.

Even though the fat supplementation has beneficial effects on both milk CLA and environmental sustainability, the excess of addition would have negative effects on the rumen fermentation and feed digestibility. Generally, the effects mainly depend on the type and the dosage of fat supplemented (21, 22). Thus, we also need to pay more attention to the effect of dietary fat supplementation on rumen function and ruminant health.

So far, a few meta-analyses have focused on the response of fat to FA composition and methane emission of cattle (23–25). Additionally, a literature review has shown the effect of medium-chain FAs on methane production, digestibility, and rumen fermentation in ruminants (22). However, no review has investigated the effects of C18 PUFA supplementation on milk CLA and methane emission associated with the microorganisms, digestibility, rumen fermentation, and BH characteristics of cattle, to our best knowledge.

This review aims to provide an overview of the existing knowledge regarding the effect of dietary fat on milk CLA and methane emission from dairy cows, while considering several metabolic processes such as rumen fermentation, BH, and microbiota response. We believe that our study makes a significant contribution to the domain because fat supplementation is a promising dietary strategy to increase the special milk fat (i.e., CLA) and to produce low carbon milk (inhibit methane emissions) by ruminants and this review provides an detailed insight into those effects.

## Effect of polyunsaturated fatty acid supplementation on conjugated linoleic acid in milk

Nowadays, people are highly interested in non-nutrients and natural nutrients that are present in foods that may have beneficial effects on humans health. CLA is one of these nutrients. CLA is an essential FA that has been shown to have anti-cancer properties in various studies in animal models (26). Principle dietary sources of CLA are dairy products and other foods derived from ruminants.

The existence of CLA in milk was first found by Booth et al. (27), who reported that a special milk fat (ultraviolet region 230 nm) was higher in the cows grazing pasture in the summer seasons than the ones without grazing. However, at that time, there was little knowledge about this special FA until Parodi (28), who first isolated *cis*-9*trans*-11 C18:2 (*c*9*t*11CLA) from milk fat. It was also believed that CLA is not normally part of a cow’s diet, and it should be a result of ruminal BH of lipids (28). Since then, more and more research studies were conducted and tried to know the synthesis of CLA and its concentration present in natural foods.

The lipid content in the dairy cow diet usually ranges from 3 to 7% on a DM basis, and most forages such as corn silage or grass silage contain C18:2 (41% of FA) or C18:3 PUFA (46% of FA) as the predominant FA. Especially, pasture-based diets fed to the dairy cow are rich in C18:3, representing 48–56% of total FA (3). It was shown that there was a significant positive relationship (r^2^ = 0.35; *P* < 0.05) between the level of pasture grass consumption and CLA content in milk fat (29). Moreover, most oil plants (e.g., soybean, sunflower, and cottonseed) and their oils are rich in C18:2 (53–69% of total FA). Peanut oil is rich in C18:1 (51% of total FA) and linseed oil contains an abundance of C18:3 (51% of total FA) (30). It has been confirmed that the addition of plant oil enriched in linolenic acid (e.g., peanut oil, sunflower oil, and linseed oil) in the diet of dairy cows can enhance the CLA concentration in milk (31). CLA was biosynthesized by rumen bacteria through isomerized C18 PUFA. CLA and other isomers occur as intermediates in the pathways of ruminal BH. Thus, it was believed that elevating the dietary C18 PUFA can increase the CLA content in milk partial through BH pathway.

## Biohydrogenation of polyunsaturatured fatty acids in the rumen

**FIGURE 1:**
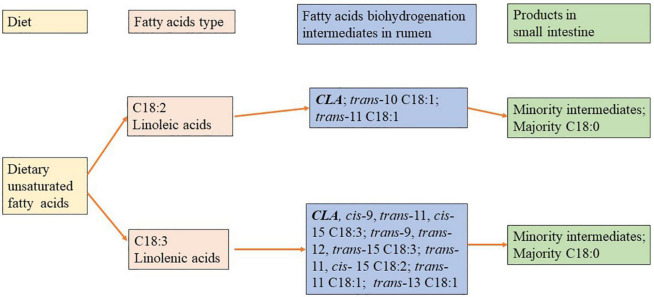
Biohydrogenation (BH) of polyunsaturatured fatty acids (PUFAs) in rumen.

Biohydrogenation is a process that occurs in the rumen in which bacteria convert UFAs to SFAs. Consequently, the FA leaving the rumen is highly saturated (32), and reduces the outflow of UFA from the rumen (Figure 1). Hence, although animals can consume large quantities of PUFAs from standard feed ingredients, most of them are converted to SFAs in the rumen and are not available for uptake into milk fat, meat, or tissues. Therefore, a thorough understanding of the extent and type of the rumen BH process is important to assess the effect of dietary PUFAs on the performance of dairy cows.

The FAs present in dairy cow feed are mainly C18 PUFAs. These dietary lipids are extensively hydrolyzed and biohydrogenated in the rumen, resulting in the production of C18:0 and a wide range of intermediates: isomers of PUFAs and MUFAs, especially *trans* and CLA (33, 34). The extent of BH of long-chain PUFAs varies significantly among different fat sources. Approximately 90–98% of C18:2n-6 disappeared after 9 h of incubation when it was supplied with the pure form (35). In contrast, Carriquiry et al. (36) reported that approximately 40–65% of the C18:2n-6 FAs residues remained after 36 h of incubation when commercial mixed fat sources were used. Furthermore, the extent of BH was strongly related to the ratio of concentrate to forage. There was a dramatic decrease in BH when the proportion of concentrate in the diets was more than 70% (37–39) because it could cause a relatively low pH, inhibiting the rate of lipolysis (40).

It has been suggested that C18:3 metabolism results in the production of a series of intermediates including *cis*9*trans*11*cis*15-C18:3, *trans*9 *trans* 12 *trans*15-C18:3, CLA, *trans*11-C18:1, and *trans*13-C18:1, whereas C18:2 metabolism results in the production of CLA, *trans*10-C18:1, *trans*11-C18:1 (41–45; Figure 1). It has been shown that these PUFAs may be converted into numerous C18:1 isomers and C18:2 (CLA) (46, 47). In addition to the nature of dietary FAs, diet composition is a key determinant of the BH intermediates composition. A high-concentrate diet added with C18:2n-6 PUFAs could result in an alteration of *trans*11-18:1 to *trans*10-18:1 in the rumen (39, 48). Additionally, it is believed that the degree of unsaturation increases with the extent of BH increases, regardless of diet. For example, Kalscheur et al. (49) reported that the extent of BH of C18:1 (54.9%) in a standard diet was less than C18:2 (74.6%), and C18:2 was less than C18:3 (83.2%). However, incomplete BH commonly occurs when cows are fed diets supplemented with C18 PUFAs. The study found that with the fat supplement in the cattle diet, intermediate *trans*-C18:1 FAs of the total C18 FA that flow to the duodenum was increased by 14.5% (from 9 to 23.5%), compared to cows fed with the control diet (49), which might have been due to sufficient PUFA being available for BH. In addition, the extent of the dietary PUFA biohydrogenation depends on FA composition and environmental conditions in the rumen such as species of the ruminal bacteria and pH of the ruminal fluid.

The determination of rumen BH in ruminants is similar to the apparent rumen organic material digestibility assessment (50). Hence, ruminal BH of C18 PUFAs in the diet is estimated as the residue of PUFAs relative to feed intake. It includes two different processes: (1) the hydrolysis of esterified fat and (2) the isomerization of 18:2n-6 and 18:3n-3 (51). This model could be used for both *in vivo* flow data and *in vitro* batch measurements (52):


(1) BHPUFA(%)=100×(PUFA-0hPUFA)th/PUFAh0

where, PUFA_0*h*_ is PUFA intake or *in vitro* PUFA supply (g) before incubation, PUFA_*th*_ is the duodenal or omasal flow of PUFA (g) or PUFA (g) recovered in batch *in vitro* culture bottle after t (h) of incubation.

However, this mechanism does not provide detailed knowledge of the nature of the accumulating CLA or other intermediates. Rumen FA metabolism is a multistep process that requires measures to assess the conversion efficiency of each step. Calculations are complex because of the movement of FAs among pools (53–55). Additionally, the limited knowledge of possible (secondary) BH pathways and the limited amount of data in most experiments limit the development of multi-compartment models including all the intermediates (54). Consequently, these approaches exclude the identification of possible shifts in BH pathways, which is beyond the scope of this study.

We concluded that the addition of C18:2 or C18:3 PUFA in the diet of dairy cattle can increase milk CLA partially through the ruminal BH process pathway. However, the detailed knowledge of the formation mechanism in CLA and its conversion efficiency is still unclear.

## Endogenous synthesis of conjugated linoleic acid

Recently, it has been argued that only a small portion of CLA escapes BH in the rumen and flows into the milk. The major portion of *c*9*t*11 CLA in milk comes from the endogenous synthesis in the mammary gland via a pathway related to the desaturation of vaccenic acid by the Δ^9^-desaturase enzyme. Studies show that the abomasum of a cow infused with vaccenic acid (12.5 g/d) for 3 days, can increase the CLA content of milk fat by 40% (56). Additionally, inhibition of the Δ^9^-desaturase enzyme could dramatically decrease the CLA content of milk fat (60–70%) (57). For lactating cows, the highest activity of the Δ^9^-desaturase enzyme occurs in the mammary gland. Moreover, the endogenous production of CLA in the mammary tissues of cows fed in pasture cannot be removed (56). However, there is limited research exploring the factors that regulate Δ^9^-desaturase activity in the tissues of cows and very little knowledge of the effect of the level of Δ^9^-desaturase on various tissues regarding CLA synthesis.

Even though the majority of the CLA from endogenous synthesis is not from the rumen BH, it is firmly believed that the addition of oils rich in C18:2 and C18:3 FA can increase the production of CLA in milk, since the rumen BH could enhance the vaccenic acid content which potentially being the additional substrate for the endogenous synthesis of the *c*9*t*11CLA.

In summary, manipulating the diets of the cow by fat supplementation can increase the CLA contents of milk. However, it is still challenging to analyze and detect all of the isomers of CLA. It would be significant for future research to investigate the biological role of individual isomers.

## Effect of polyunsaturated fatty acid supplementation on digestion of nutrients in rumen

The effects of PUFAs on ruminal digestion of nutrients may vary, and the amount of forage used in the diet can be considered a key factor (1). Interactions between the degradation and passage rates determine the extent of digestion in the rumen and the amount of undegradable material in the feces. The effect further depends on the form of PUFA (n-6 or n-3) and whether it is freely or partially protected when it enters the rumen.

From the standpoint of rumen carbohydrate fermentation, BH is a favorable process because it reduces the potential negative effects of UFA on rumen fermentation of fiber. This suggests that UFAs are toxic to many species of rumen bacteria, especially those involved in fiber digestion (2). A decrease in DM, organic matter (OM), and neutral detergent fiber (NDF) digestibility was observed in dairy cows fed linseed oil at relatively high levels (5.8 or 7% of DM) (58, 59). However, several studies did not find a negative effect of dietary C18 PUFAs on fiber degradation, especially when the amount of plant oil supplementation was relatively low (≤5% of DM). For instance, previous studies reported that the addition of flaxseed oil (enriched in C18:3 PUFA) at around 2.4% DM increased ether extract (EE), and NDF digestibility (60, 61). Similarly, Pi et al. (62) observed that plant oil (enriched in C18:3 PUFA) supplementation at 4% DM in the diet increased the digestibility of NDF and DM. Additionally, a previous study reported that dietary supplementation of plant oils at 3% DM enhanced the digestibility of NDF and EE using a rumen-simulation technique (63). Supplementation with different sources of plant oil (2 or 5% of DM), enriched in C18:2 PUFA in diets, does not affect NDF degradability (64, 65). These results highlight the fact that the effect of dietary oil supplementation on the digestibility of nutrients in dairy cows largely depends on the type and dosage of the oil being used.

It has been shown that oil supplementation did not shift the crude protein (CP) digestibility (62). However, Atikah et al. (66) reported that the addition of oil increased the digestibility of CP because fat can be used as a source of energy for rumen microorganisms to convert feed protein into microbial protein, which is more digestible. Additionally, increased CP digestibility may be explained by the reduction in microbial degradation by protozoa, which in turn increases the amount of protein available in the post-gastrointestinal tract (67).

A diet supplemented with plant oil has a higher apparent digestibility of EE (66, 68). Bauchart et al. (69) explained this by revealing that diets rich in dietary fats tend to have a higher hydrolysis percentage in the rumen than the conventional diet. It has also been shown that lipases related to rumen lipid hydrolysis can be more active in diets with high fiber and protein contents (70). Jenkins (71) reported that PUFAs with an increasing number of double bonds had higher digestibility.

**FIGURE 2:**
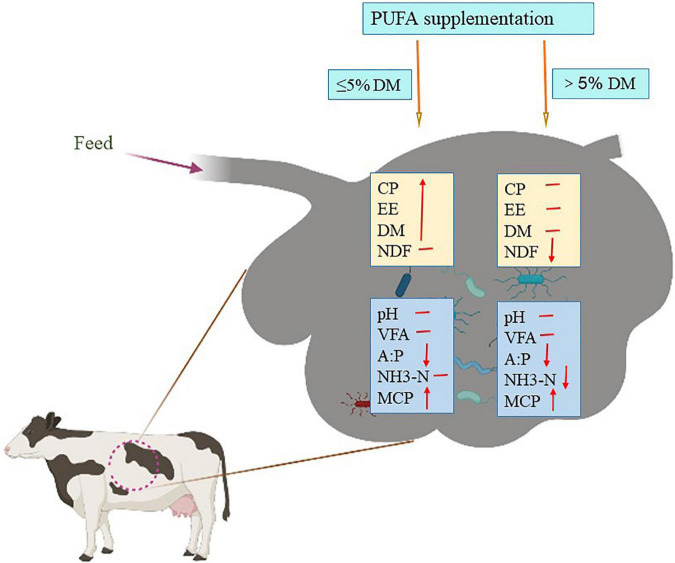
Effect of polyunsaturatured fatty acid (PUFA) supplementation on digestion of nutrients and fermentation in rumen. DM, dry matter; CP, crude protein; EE, extract ether; NDF, neutral detergent fiber; VFA, volatile fatty acid; A:P, the ratio of acetic acid and propionic acid; NH3-N, ammoniacal nitrogen; MCP, microbial proteins. ↓: Represents the decreasing effect; ↑: represents the increasing effect; —: represents no effect.

In conclusion, the increase in dietary PUFAs did not have a negative influence on NDF degradability if the dosage of plant oil was relatively low (≤5% of dietary DM), and it may increase the apparent digestibility of CP and EE regardless of the dosage level (Figure 2).

## Effect of polyunsaturatured fatty acid supplementation on fermentation parameters in the rumen

Ruminants acquire energy from the feed materials through microbial fermentation. During the fermentation process, energy is released as adenosine triphosphate, which is used to fuel the different activities of ruminal microorganisms. Energy levels can be increased by supplementing animals with dietary fat, an approach that has been widely used. However, the reported effect of oils on rumen fermentation varies dramatically among studies, depending on the concentration, origin, saturation degree, FA composition of the fats used, and the nutrient composition of the diets (22, 72; Table 1 and Figure 2).

**TABLE 1:** Effects of different polyunsaturated fats supplementation on rumen fermentation in ruminants.

Fat type	Dosage (DM basis)	Diet	Effects	References
High C18:2n6	5% of linoleic acid-rich of total substrate	60:40 forage to concentrate (*In vitro*)	No changes in total VFA concentrations and ratios of acetic to propionic acid.	(90)
High C18:2n6	4% of soybean oil or sunflower oil of concentrate	50:50 forage to concentrate (*In vivo*)	No changes in ammonia and VFA concentrations.	(98)
High C18:2n6	6% of soybean oil of TMR	The ratio of forage to concentrate was not mentioned (*In vivo*)	Oil supplement significantly (*p* < 0.05) decreased ammonia concentration in goats, but no changes were observed in total VFA concentration.	(66)
High C18:2n6	3% of soybean oil of TMR	60:40 forage to concentrate (*In vivo*)	Total VFA was increased (*p* < 0.05) but no shifts in proportions of individual VFAs.	(207)
High C18:2n6	2% of soybean oil of total DMI	1:2 concentrate to milk production (*In vivo*)	No changes were observed on ammonia concentration.	(97)
High C18:2n6	5% soybean oil of concentrate mixture	1.5% BW of concentrate and free access to rice straw (*In vivo*)	Ammonia concentration, and VFA concentration in goat were decreased (*p* < 0.001) compared with control treatment.	(96)
High C18:2n6	4% of soybean oil of total DMI	60:40 forage to concentrate (*In vivo*)	Ruminal pH did not differ; total VFAs were lower (*p* < 0.05) than control group; ammonia concentration increased (*p* < 0.05).	(88)
High C18:2n6	3% of linoleic acid of total substrate	50:50 forage to concentrate (*In vitro*)	Total VFAs were decreased (*p* < 0.05).	(94)
High C18:2n6	3% of linoleic acid of total substrate	70:30 forage to concentrate (*In vitro*)	Total VFAs were higher (*p* < 0.05) than control group; no effect on acetate to propionate ratios.	(88)
High C18:3n3	2, 3, or 4% of linseed oil of total DMI	50:50 forage to concentrate (*In vivo*)	No changes on apparent total-tract digestibility of nutrients. Ruminal ammonia, pH, and total VFA concentrations did not differ.	(86)
High C18:3n3	2.2% of flaxseed oil or 2.7% Ca-salts of flaxseed oil of total DMI	Calves starter feed (*In vivo*)	No changes on ruminal pH, individual VFA and total VFAs concentration.	(87)
High C18:3n3	10% of flaxseed oil of total substrate	Diet consisting of hay, barley, and sugar beet molasses (60:30:10) (*In vitro*)	Increasing (*p* < 0.05) the molar percentage of propionate.	(92)
High C18:3n3	4% of flaxseed oil of total DMI	60:40 forage to concentrate (*In vivo*)	Ruminal pH did not differ; total VFAs were lower (*p* < 0.05) than control group; ammonia increased;	(88)
High C18:3n3	4% of linolenic acid of total substrate	50:50 forage to concentrate (*In vitro*)	Total VFAs were decreased (*p* < 0.05).	(94)
High C18:3n3	4% of flaxseed oil or rubberseed oil of total DMI	Around 50:50 forage to concentrate (*In vivo*)	Increasing (*p* < 0.05) the proportion of propionate.	(62)
High C18:3n3	3% of α-linolenic acid of total DMI	70:30 forage to concentrate (*In vitro*)	Total VFAs were increased (*p* < 0.05); no changes on acetate to propionate ratios.	(95)
High C18:3n3	6% of linseed oil of total DMI	High-concentrate TMR (*In vivo*)	Total VFAs concentration did not differ, ammonia and butyrate concentrations were decreased (*p* < 0.05), propionate concentration was increased (*p* < 0.05).	(91)
High C18:3n3	2, 3, or 4% of linseed oil of total DMI	50:50 forage to concentrate (*In vivo*)	Ruminal pH, ammonia, and total VFA concentrations were not affected by treatments.	(86)
High C18:3n3	2.5% of flaxseed oil of total DMI	60:40 forage to concentrate (*In vivo*)	Ruminal pH, ammonia, and total VFA concentrations did not differ.	(89)

DM, dry matter intake; BW, body weight; TMR, total mix ration; VFA, volatile fatty acid.

### Rumen pH and volatile fatty acids

There is an interaction effect between dietary ruminal fermentation and rumen pH. Typically, rumen pH can alter ruminal fermentation and microbial growth both *in vivo* (73, 74) and *in vitro* (75–78). Previous studies have reported the effects of pH on appetite (74) and fiber digestion (76). In turn, volatile fatty acids (VFAs) production and feed type can significantly affect ruminal pH. It has been demonstrated that pH decreases as the concentration of dietary starch increases because of rapid acid accumulation (79, 80). Additionally, it has been reported that the pH could be shifted by the concentrate-to-forage ratio and particle length (81, 82). However, FA supplementation seems to have little effect on rumen pH (83–89; Figure 2).

Szumacher-Strabel et al. (90) found that supplementing 5% high-LA oil (C18:2 PUFA) did not disturb rumen fermentation, concentrations, and ratios of acetic acid to propionic acid. This also suggests that the supplementation of dairy cows with high-ALA oil (C18: 3 PUFA) did not affect ruminal pH or total VFA concentrations (86). Similarly, other studies have also reported that the addition of C18 PUFAs does not affect total rumen VFA concentrations (66, 87, 89, 91). Furthermore, studies have shown that feeding vegetable oils rich in PUFAs can modify the rumen fermentation pattern by increasing the molar percentage of propionate (62, 88, 91, 92). For example, Doreau and Chilliard (93) reported that high-LA oils could inhibit rumen fermentation by inhibiting VFA production and decreasing the acetate-to-propionate ratio.

*In vitro* studies have also shown that the addition of C18 PUFAs significantly altered the ratio of acetate to propionate (94, 95). A recent *in vivo* study demonstrated that a diet supplemented with 4% oil enriched in C18:3 PUFAs improves the total tract apparent digestibility of nutrients (DM, NDF, and EE) and changes the rumen fermentation pattern by increasing the proportion of propionate (62). Additionally, an increase was observed in the valerate concentration after supplementing oil enriched in C18:2 PUFAs to *in vitro* goat diets (90). These results suggest that dietary C18 PUFA supplementation, in general, does not shift rumen pH and total VFA, whereas, in most cases, C18:3 PUFAs can increase the proportion of propionate in the rumen (Table 1 and Figure 2). The addition of dietary PUFAs may explain the increase in the proportion of propionate in the rumen interfering with normal fiber digestion in the rumen, resulting in reduced butyrate and acetate (90, 92).

### Ammonia

The optimum level of ammonia that favors ruminal microbial activity in animals fed lignocellulosic fiber was between 16.5 and 37.9 mg/L. There are many conflicting results concerning ammonia concentrations when the diet is supplemented with plant oil enriched in C18 PUFAs. It has been shown that plant oil (enriched in C18 PUFA) supplementation in ruminant diets at a relatively high level (>5% of the DM) depressed the rumen ammonia concentration (66, 91, 96; Figure 2). Conversely, other studies suggest that the addition of plant oil with less than 5% of dietary DM does not affect (86, 89, 97, 98) or increase (88) the ammonia concentration in the rumen (Figure 2). This indicates that the effect of dietary PUFA supplementation on ammonia concentration depends mainly on the plant oil supplementation level in the dairy cow diet. Ammonia is usually presumed to be produced by the breakdown of bacterial protein (99), which is often associated with the presence of protozoa (100). According to Hristov et al. (101), the number of protozoa correlates positively with ammonia concentrations; reducing the number of protozoa could lower bacterial protein decomposition and result in increased microbial protein flow to the intestine. This suggests that ammonia utilization for microbial protein synthesis can be potentially increased by a higher level of supplementation of C18 PUFA enriched plant oil (Figure 2).

## Effect of polyunsaturatured fatty acid supplementation on methane emission

Methane production from ruminal anaerobic fermentation contributes to a 2–12% loss of the ingested gross energy (102) and greenhouse gas emissions (103). Therefore, for efficient animal production and global environmental protection, recently, various technologies and policies have been explored to reduce enteric methane emissions from ruminants (72, 104, 105). However, most dietary strategies proposed to reduce methane production in the rumen have negative effects on fermentation and productivity (106). For example, replacing roughage with concentrate may decrease the ruminal pH due to quick fermentation (72); the addition of essential oil potentially decreases the rumen microorganism activities (104). Few dietary interventions using these methods seem to be promising for inhibiting methane emissions while maintaining or improving ruminant performance (104, 106). The addition of fats is a dietary method known to reduce enteric methane emissions (72, 107). Additionally, fat supplementation can increase the energy density of diets and enhance the energy status of high-yielding lactating dairy cattle (108). The addition of C18:3n-3 PUFAs can also increase the concentration of CLA and C18:3n-3 PUFAs in milk and meat, which is beneficial to human health (109).

### Methane inhibitors

Many methane inhibitory additives have been proven to affect methane production in ruminants, either by sequestering hydrogen (110, 111) or by affecting rumen microflora (104). These compounds mainly include halogenated methane analogs, ionophores (103) (i.e., antibiotics), and biologics (112) (i.e., bacteriocins, viruses, yeasts). Unfortunately, from the food safety viewpoint, there is an increasing perception that these chemical compounds are unacceptable for general use in the agricultural industry (113).

A review emphasized that short-term nutritional strategies could reduce methane production, including supplementation with tannins, fiber-digesting enzymes, and yeast cultures (72). Additionally, more promising interventions, such as nisin and hydrogen-precursor substances (i.e., fumarate and malate), could also be considered acceptable for general use in agricultural settings. It has been reported that nisin can decrease methane production by approximately 36% *in vitro* (114). Similarly, Newbold et al. (115) found that fumarate supplementation could decrease methane emission by 17%. Furthermore, hydrogen-precursor resources such as fumarate, which are natural intermediary products in rumen metabolism, can cause relatively fewer ethical and food safety issues. Therefore, the hydrogen-precursor resource is an alternative approach for reducing methane emissions, and it does not have a negative effect on animal productivity and product quality. However, these substances are relatively expensive and implementation of this type of technology is difficult.

### Effects of different dietary fats on methane emission

Most studies have shown that dietary fat supplementation can depress enteric methane emissions, although the extent of inhibition varies (21, 116). Particularly, many studies have reported that supplementation with C18 PUFA-rich oils inhibits methane production *in vitro* (117, 118) and *in vivo* (119) with the extent varying depending on the unsaturation degree and inclusion level (120). For example, it has been shown that the diet supplemented with sunflower seeds (rich in C18:2n-6 PUFAs) reduced methane emission in lactating ewes and cows by 27 and 10% (121), respectively, while the addition of linseed oil (rich in C18:3n-3 PUFAs) resulted in an emission decrease of 10% in lambs and 18% in cows (110). Moreover, rapeseed supplementation (rich in C18:1 UFAs) decreased methane production by 16% in cows and 19% in lambs (122). Machmüller et al. (122) found a 27% reduction in methane emission *in vitro* in lambs fed sunflower seeds. A decrease in methane production of 12–64% in cows was observed when the diet was supplemented with different linseed oils (58). It suggests that every 1% increase in dietary fat supplementation can cause a decrease of 13.4 g/day (or 4.3%) in methane emissions (123). The diversity of PUFA contents used in these studies may partially explain the variations in their effects on reducing methane production in ruminants.

**FIGURE 3:**
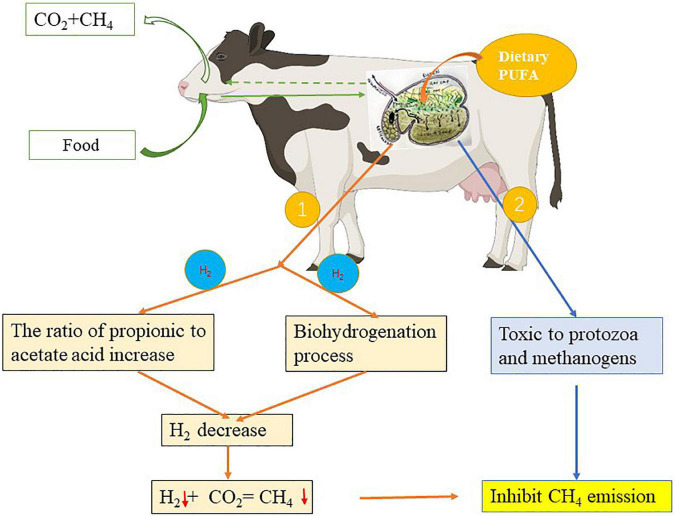
Effect of polyunsaturatured fatty acid (PUFA) supplementation on methane emission. ①: The pathway of PUFA use hydrogen for the biohydrogenation (BH) reaction and propionic acid production associated with the decrease in methane emission, ②: the pathway of PUFA directly affects the methanogenic activity of the rumen microbiota thus reducing methane emission. ↓: Represents the decreasing effect.

Unsaturated fatty acid may use hydrogen for the BH reaction and propionic acid production is associated with a decrease in methane production (Figure 3). However, only a small quantity (1%) of total metabolizable hydrogen is used in this endogenous UFA process relative to that used for the reduction of CO_2_ to methane (48%), bacterial cell synthesis (12%), and VFA synthesis (33%) (124). Therefore, the main reason for the reduction in methane production when UFA was added may not be due to their BH reaction (119), but presumably a direct effect on the rumen methanogenesis by microorganisms (Figure 3). Dietary PUFAs inhibit methanogenesis by reducing the metabolic activity and rumen methanogen population (121). However, oil supplementation could decrease methane emission in defaunated animals (112), indicating that the removal of methanogens associated with protozoa is not the only cause of the depression of methanogenesis. Zhang et al. (120) and Prins et al. (125) have shown the direct toxicity of C18 PUFAs on methane production. It was confirmed that PUFAs also inhibit methane production by reducing the number of methanogens (126, 127). This suggests that C18 PUFAs reduce methane production by inhibiting ciliate protozoa and methanogenic bacterial populations.

Daily methane production from dairy cows varies among studies 400–500 g/d (methane: 1 L ≈ 0.7143 g) depending on the DM intake (DMI), milk yield, and diet type, and the methane production ranged from 18 to 25 g/kg DMI and 10 to 24 g/kg milk (116, 128, 129). PUFAs are observed to be more toxic to rumen microbes than MUFAs, and the increasing inhibitory effect of fat on methane production increases with the increasing degree of unsaturation of FA (24). Moreover, UFA is supposed to be more potent than SFA in inhibiting methane-producing processes (116).

It has been reported that there was 0.8–5.5% reduction in methane production in the fat supplementation group compared with that of the control group (129). This suggests that the average levels of reduction in methane with the addition of fats were 4.7–5.1% (21, 130). Additionally, a previous study showed that the lactation stage does not affect the production of methane in g/d (131). Similarly, Cammell et al. (132) found that methane reduction as a percentage of digestible energy increased from week 6–24 post-calving as the DMI decreased. It has also been found that daily methane production increased during the first 10–12 weeks of lactation, which may be due to the expected increase in the DMI and milk yield (133, 134). From week 10 onward, and in subsequent lactation, Garnsworthy et al. (134) estimated that methane production began to decrease. Furthermore, it has been shown that the effect of fat supplementation on methane production was persistent during the lactation stage, although they only measured methane production up to week 16 (128, 133, 135). It has been shown that the whole cottonseed causes a persistent reduction in methane production (133, 135). Similarly, a study found that fat influences methane production throughout lactation. This effect on methane production is persistent, and methane production increases with daily milk yield (129).

Additionally, according to Patra (123), methane production could be estimated by dietary fat content with a high degree of prediction (R^2^ = 0.64), which is higher than NDF (R^2^ = 0.42) and non-fibrous carbohydrate (NFC, R^2^ = 0.43) as sole predictors. Concentrations of NDF and NFC (R^2^ = 0.79), NDF and fat (R^2^ = 0.73), NFC and fat (R^2^ = 0.95), and NDF, NFC, and fat (R^2^ = 0.80) could improve the methane prediction compared to single predictors. This suggests that using the concentrations of NFC and fat is the best approach to predict methane production, although the high prediction accuracy does not seem from a biological perspective, considering data were collected from several sources.

These results suggest that C18 PUFA supplementation can inhibit methane emission in the rumen by decreasing the number of ruminal protozoa and methanogens bacteria. However, the extent of the depression is majorly dependent on the unsaturation of PUFA, the amount of the PUFA addition, and the dietary type, but not on the lactation stage.

## Rumen microorganisms’ response to polyunsaturatured fatty acids

In ruminant animals, most of the digestive and metabolic processes, such as organic matter (OM) fermentation, lipolysis, BH, and methane emissions, are involved in ruminal microbiotic activity, including bacteria, fungi, protozoa, and archaea. Despite a highly stable function because of functional resilience and redundancy of the ruminal microbial ecosystem (136), the rumen microbiota shows great variations between individuals (137) and can be interfered with abrupt or major dietary alterations, such as starch or fat content.

### Effects of different fats on protozoa, bacteria, and fungi

Early studies regarding oil supplementation in ruminant diets were primarily related to its toxicity toward protozoa. A dramatic decrease in the protozoan population occurred when the diet was supplemented with three different C18 UFAs: OA (C18:1), ALA (C18:3), and LA (C18:2) (120). The inhibition of ruminal protozoa by C18 UFAs was also reported by Hristov et al. (138). The inhibitory effects may be attributed to the ability of protozoa to absorb lipids while lacking lipolytic activity, resulting in the accumulation of free FAs in the cell, eventually leading to cell death (139–141). This suggests that increasing the degree of unsaturation decreases the protozoan population, but this alteration can be difficult to evaluate because of the random and individual variations, which partially explain the inconsistent data over experiments (142).

Dietary plant oil supplementation may also affect the rumen bacteria. The suppressive effect of plant oils on the growth of ruminal microorganisms has been largely investigated in pure cultures of rumen strains (143, 144), mainly focusing on bacteria known to contribute to amylolysis, fibrolysis, and FA metabolism. A study showed that OA (C18:1 UFA) is far more depressive than palmitic (C16:0) and stearic acids (C18:0) in the population of most fibrolytic bacteria but stimulates the growth of *Selenomonas ruminantium* and *Prevotella ruminicola* (143). This is also supported by Henderson (145), who reported that bacteria (*S. ruminantium*, *Anaerovibrio lipolytica*, *P. ruminicola*, and *Megasphaeraelsdenii*) were not adversely affected by OA, while *B. fibrisolvens* and *Ruminococcus*, which are mainly related to acetate and butyrate production, were inhibited by OA. Further studies on pure strain cultures showed that LA inhibits the growth of *B. fibrisolvens* A38 at very low concentrations (4 mg/L) (146), and that the adverse impacts of ALA on *B. fibrisolvens* are greater than LA because of a longer lag phase in growth cultures (14, 147). This suggests that the degree of FA unsaturation plays an essential role in affecting the growth of fibrolytic bacteria in rumen culture. These results are consistent with *in vivo* studies, showing that the addition of vegetable oils to ruminant diets can cause different responses in rumen microbial populations and ruminal fermentation, mainly depending on the quantity and type of fat supplemented, as well as diet composition (148–150). For example, it has been shown that cows fed diets supplemented with plant oils (LA and ALA) have a lower number of cellulolytic bacteria and protozoa, but a higher number of proteolytic bacteria than cows fed the control diet, especially when the oil is rich in ALA (88). Furthermore, a diet supplemented with over 8% of total fat can reduce DMI and fiber digestibility in the rumen (86), and a higher amount of C18 PUFAs causes greater inhibitory effects on the rumen bacterial population (71, 151). In general, diets for ruminants have a low percentage of fat (∼2–3%) due to the nature of the ingredients used (152). This indicates that oil supplementation levels lower than 5% of the diet (DM basis) generally did not affect rumen fermentation. The inhibitory effect of high-level fat supplementation on feed degradation was mainly due to limiting bacterial growth and reducing bacterial contact with the feed particles. This may be due to oil supplementation causing destructive interference by the “coating effect” (coating the bacterial cell and feeding particle surface) (91). However, the “coating effect” theory is still under debate. This theory still requires extensive testing and agreement within the scientific community.

The effects of oil supplementation on rumen fungi have rarely been reported, but it is still recognized that the addition of PUFAs can affect fungi. In pure cultures, LA alters the growth of the fungus *Neocallimastix frontalis* (144). A previous *in vivo* study revealed that LA supplementation adversely affected the order Neocallimastigales, whose abundance and diversity was reduced by soybean oil supplementation (153).

In general, the supplementation of PUFA can decrease the number of protozoa and bacteria, and potentially reduce the fungi abundance. These effects on microorganisms mainly depend on the quantity and type of fat supplemented. In addition, there is still limited knowledge about the effect of PUFA on fungi, and the further investigation using the new technology is necessary.

### Microorganisms response to polyunsaturatured fatty acids in lipolysis

Lipolysis occurs rapidly after food intake in ruminal animals (154, 155). The forage ingested by ruminants consists of low concentrations of triacylglycerol, mainly sulfo-lipid, galacto- and phospholipids (33). Plant tissues are rich in galactopolipases and phospholipases. These lipases remain active once ingested for up to 5 h in the rumen, indicating that the plant material itself may contribute to ruminal lipolysis in grazing animals (156). Lee et al. (157) and Van Ranst et al. (158) also suggested that plant lipases contribute to the overall ruminal lipolysis. Unfortunately, none of these studies have compared plant lipases activity with that of ruminal microorganisms. Nevertheless, microbial lipases are commonly believed to be more important than plant enzymes (155).

Despite the abundant diversity of ruminal microorganisms, bacteria are considered to be the most active in lipolysis. This view was supported by experiments with ciliate-free sheep, in which the hydrolysis of phosphatidylcholine was high (155). *Anaerovibrio lipolyticus* is a well-known triacylglycerol-hydrolyzing bacterium (159, 160), whereas, *A. lipolytica* is dominates the ruminal lipase activity in animals receiving concentrated feed and lacks the ability to hydrolyze galactolipids and phospholipids. Therefore, other lipolytic species are expected to predominate in the rumens of grazing animals. It has been confirmed that galactolipids and phospholipids could be hydrolyzed by some strains *B. fibrisolvens* (161) and *Butyrivibrio*-like species (161, 162). *Butyrivibrio* spp. seemed to contain phospholipase A, phospholipase C, phosphodiesterase, and lysophospholipase activities typical of mixed rumen contents (163). In fact, these bacteria are also capable of biohydrogenation of UFA, which indicates that the two properties are linked in a way that benefited their “biochemical economy” (163).

However, the lipolytic activity of protozoa has not been extensively studied. Wright (164) reported that *Epidinium* spp. accounted for 30–40% of lipolytic activity in the rumen. *Epidinium ecaudatum* has galactosidase activity and is capable of releasing galactose from galactolipids (165). Another protozoan species, *Entodinium caudatum*, was observed to have phospholipase activity (166) which appears to be more relevant to the internal environment of the protozoa than to the lipolysis of dietary lipids. Additionally, a previous study suggested that the lipolytic activity in protozoa fractions was higher due to the activity of the bacteria that the protozoa had ingested than that of the individual protozoa itself (167).

Recent data have shown that lipolytic activity is involved in other rumen bacteria, including *Clostridium*, *Propionibacterium*, *Selenomonas*, and *Staphylococcus* genera (168), as well as lipase of a *Pseudomonas aeruginosa* strain (169). Recent studies have also used advanced technologies, such as metagenomics, pyrosequencing, sequence databases, and analogous technologies to improve our knowledge of the lipolytic activity of FAs in the rumen. For example, Liu et al. (170) identified two lipases with high affinities for C16 and C18 FA from a metagenomic library of cow rumen. However, their significance to the overall rumen community is uncertain. Indeed, several lipase sequences need to be analyzed from the metagenome to understand the nature of lipolytic enzymes in the rumen. However, it might be premature to study the metagenome before interrogating the genome of known lipolytic species. Therefore, to investigate lipolytic enzymes, pure culture studies using metagenomics should be combined with future research.

### Microorganisms response to the polyunsaturatured fatty acids in the rumen biohydrogenation process

It is well-known that the BH of PUFAs is a characteristic biochemical reaction driven by ruminal microbiota. Pioneering studies have reported that rumen microorganisms are capable of hydrogenating C18:1, C18:2, and C18:3 PUFAs to form C18:0 SFAs (171–173).

Protozoa account for approximately 50% of the ruminal microbial biomass and about 75% of the microbial FAs present in the rumen originate from protozoa (174). Therefore, protozoa can be an important source of vaccenic acid (the processor of CLA in the endogenous synthesis process) and CLA. However, the protozoa directly performing BH in the rumen are yet to be determined. It has been shown that LA composition does not alter when incubated with protozoa alone (175). Interestingly, protozoa are capable of ingesting bacteria, and the bacterial BH may take place within the host protozoa (32) which explains the high concentration of BH intermediates (175) produced by protozoa. Rumen fungi have also been reported to have limited ability to BH LA (32, 144) to produce OA, and all BH reactions were completed within 24 h of incubation with fungi (176). Therefore, bacteria play a key role in PUFA BH (32).

It has been shown that *B. fibrisolvens* can convert LA to *trans* C18:1 but not to C18:0 (177). Moreover, *B. fibrisolvens* also hydrogenates LA to *trans* intermediates (178). Maia et al. (144) found that *B. hungatei* can also convert LA to OA. Additionally, Paillard et al. (160) and Hussain et al. (179) demonstrated that LA metabolism varied among *Butyrivibrio* isolates, and a large number were capable of metabolizing LA to OA. Minor differences in BH pathways among strains were also reported by Fukuda et al. (147), who found that *MDT-5*, *MDT-10*, and *A38* strains could metabolize LA to *trans-*11 *cis-*13 CLA, OA, and *trans-*11 *cis-*15 C18:2, respectively.

The first step of BH was isomerization by *B. fibrisolvens*, producing *trans*-11 UFA. Kepler and Tove (178) reported that *B. fibrisolvens* D12 isomerase is localized in membranes or tightly attached to bacterial membranes. Its substrates are FA with a free carboxyl function and double bonds on *cis*-9 and *cis*-12 carbons (180). Other bacteria could isomerize LA to *cis-*9 *trans-*11 CLA, such as *Bifidobacterium*, *Clostridium*, *Pseudobutyrivibrio*, *Lactobacillus*, *Propionibacterium*, *Eubacterium*, *Roseburia*, *Enterococcus*, and *Pediococcus* genera (169, 181, 182). Lactic acid bacteria produce CLA via a hydration-dehydration process using hydroxy FAs as an intermediate (183). UFA hydration in the rumen is mainly due to *Streptococcus bovis*. Several strains of *Streptococcus*, *Staphylococcus*, *Lactobacillus*, *Enterococcus*, and *Pediococcus* can catalyze this reaction (184). *Ruminococcus albus* F2/6 (185) can metabolize C18:2 and C18:3 to C18:1, whereas their relative activity in the rumen remains unclear (32). Bacterial strains identified as *M. elsdenii YJ-4 and M. elsdenii T81*, could convert C18:2 to *trans*-10 *cis*-12 CLA in the rumen of a cow fed a high-starch diet (186).

The final step of the BH process is to convert the BH intermediates to SFAs. It was found that two bacterial species belonging to the genus *Fusocillus* can reduce C18:1 FA to C18:0 (33). In addition, a strain of *Butyrivibrio*, which is phenotypically similar to *Fusocillus* can complete the BH of both C18:2 and C18:3 to C18:0 (187).

Other strains of the *Butyrivibrio* group are also capable of producing stearic acid from LA and are involved in the decrease of OA (188). OA reductase differs from CLA reductase because many bacteria that convert CLA to OA do not reduce OA to stearic acid. Furthermore, *B. proteoclasticus* can reduce both CLA and VA, and the two reductases used are probably different as they are not similarly affected by lactic acid (144).

Several studies have also been conducted to evaluate the relationship between BH and ruminal microorganisms *in vivo* and *in vitro* by inoculating with bacteria and measuring BH products and adding bacteria or observing the effect of dietary supplements on BH products and bacterial abundance. A previous *in vivo* study showed that *Fibrobacteriaceae* family had the highest and most significant correlation with FAs related to the BH process of C18:3 (189). Inoculation of *B. fibrisolvens* in the rumen of the goats and supplementation of their feed with LA increased VA and total CLA concentrations in the rumen fluid, which indicates that this bacterium is involved in the BH process *in vivo* (190). The other major bacterial species linked to the BH metabolism belong to the *Butyrivibrio* group, which includes the genera *Butyrivibrio* and *Pseudobutyrivibrio* (191). Additionally, BH is not a nutritional process but a detoxification process in bacteria; therefore, the abundance of bacterial BH process is probably more strongly related to its high energy substrate (toxic for bacteria) than to UFAs. Although sequencing is an efficient method to identify bacterial abundance and diversity, it has several shortcomings. Most studies do not identify species unequivocally and as the BH bacteria are closely related (192–194), identifying the different species accurately would be difficult. The measurement of RNA concentration might be more indicative of the microbial taxa responding to the dietary challenge.

### Microorganisms response to polyunsaturatured fatty acids in methane emissions

Methane, a natural byproduct of ruminal fermentation, is mainly produced by archaea by utilizing CO_2_ and H_2_, which originate mostly from the fiber degradation activity of bacteria, protozoa, and fungi (195). A previous study suggested that even a small amount of H_2_ accumulated in the rumen can inhibit sugar oxidation, hydrogenase activity, and VFA conversion (196). The activity of hydrogen-utilizing methanogens in the rumen reduces the end-product inhibition of H_2_, thus allowing more rapid fermentation of feed (197). The capture of H_2_ produced by other microbes is referred to as interspecies H_2_ transfer, which usually occurs between archaea and other microbial species (111). Therefore, methane production can be affected by the abundance of various microbial species.

There is limited understanding of the effects of fats on ruminal microbiota, as well as the associated methane production. Several studies revealed that fat supplementation inhibited some ruminal microorganisms possibly due to their direct toxicity toward protozoa and archaea (198, 199). Additionally, the BH of PUFAs can act as an alternative H_2_ sink, thus selectively reducing the H_2_ for archaea. However, only approximately 1–2% of the total H_2_ available can be utilized by this pathway (200, 201). The effectiveness of lipids in mitigating methane may be related to their FA composition (202).

A mixed culture study (120) and a pure culture study (125) showed that C18 PUFAs could reduce methanogen growth. As some methanogens live in association with protozoa (203, 204), it was expected that the reduction in protozoa would also reduce methanogens. This also suggests that the sensitivity of protozoa to C18 PUFAs is much greater than that of methanogens (120). Approximately 10–20% of methanogens that live in association with protozoa (203) would decrease along with protozoa, whereas free-living methanogens might not be affected by C18 PUFAs to the same extent. Moreover, it suggests that LA and ALA decrease the methanogen population relative to total bacteria mainly because of the reduction of ruminal H_2_ availability (120). Methanogens survive on H_2_ in the rumen and compete with propionate-producing microorganisms for this substrate. This is also supported by Sun et al. (205), who reported that the addition of raw flaxseed (rich in C18:3 PUFAs) tends to increase the concentration of propionate during *in vitro* fermentation and decrease the methane as associated with the relative abundance of *Methanobrevibacter* decline and *Methanobrevibacter* is considered the predominant protozoa-associated methanogen (206). Therefore, an increase in the molar proportion of propionate with the dietary C18 PUFAs supplements resulted in lower availability of H_2_ for methanogens.

It has been found that the effects of oilseeds enriched with C18 PUFAs and those of the removal of fungi appeared to be widely synergistic, resulting in a final methane suppression up to 45% per unit of digestible OM, compared to intact rumen liquid incubated with the basal diet as the sole substrate (195). This indicated that C18 PUFAs exert indirect effects on methane suppression by decreasing H_2_ production and inhibiting the growth of cellulolytic ruminal fungi (120, 144). Moreover, it has been shown that ALA with three double bonds reduces methane emissions mainly by its toxicity against bacteria or archaea, whereas LA with two double bonds exerts an inhibitory effect on fungal growth (144, 195). The reason why ALA and LA have different effects on fungal growth is still unclear.

## Conclusion

Concerning rumen physiological activity, a throughout understanding of the symbiotic relationship among microorganisms is important for predicting the response of microorganisms to C18 PUFAs. The addition of PUFAs in dairy cow diets would increase the CLA contents of milk and other dairy products, and many rumen bacterias play a key role in this pathway that isomerizes LA to *cis*-9*trans*-11 CLA, such as *Bifidobacterium*, *Clostridium*, *Pseudobutyrivibrio*, *Lactobacillus*, *Propionibacterium*, *Eubacterium*, *Roseburia*, *Enterococcus*, and *Pediococcus* genera.

Additionally, the inhibitory effect of dietary fats supplements on methane emissions seems to be 2-fold. Firstly, it acts by inhibiting the activity of the methanogenic bacteria associated with limited protozoa and fungi in the rumen. Secondly, it acts as an H_2_ consumer. Consequently, dietary fat supplementation results in a decrease in the digestibility of fiber and acetate, and an increase in the propionate to acetate ratio, finally resulting in a decrease in H_2_ production and thereby methane emission. This means that dietary fats supplementation also can produce milk with lower greenhouse gas emissions.

Even though the effect of PUFA on microorganisms have been extensively studied, the underlying mechanism remains unclear. Further studies are necessary to test the existing hypothesis regarding such an effect. Omega-3 is a type of PUFA considered beneficial to human health. It was not discussed in this review but it would be meaningful to be addressed in future studies.

## Author contributions

XS and WW: conceptualization. XS: writing—original draft preparation. YW, XM, and SL: writing—review and editing. WW: supervision. SL: funding acquisition. All authors have read and agreed to the published version of the manuscript.
